# Advanced glycation end products measured by skin autofluorescence and subclinical cardiovascular disease: the Rotterdam Study

**DOI:** 10.1186/s12933-023-02052-7

**Published:** 2023-11-28

**Authors:** Jinluan Chen, Banafsheh Arshi, Komal Waqas, Tianqi Lu, Daniel Bos, M. Arfan Ikram, André G. Uitterlinden, Maryam Kavousi, M. Carola Zillikens

**Affiliations:** 1 Department of Internal Medicine, Erasmus MC, University Medical Center Rotterdam, Doctor Molewaterplein 40, 3015GD Rotterdam, The Netherlands; 2 Department of Epidemiology, Erasmus MC, University Medical Center Rotterdam, Rotterdam, The Netherlands; 3 Department of Radiology & Nuclear Medicine, Erasmus MC, University Medical Center Rotterdam, Rotterdam, The Netherlands

**Keywords:** Advanced glycation end products, Atherosclerosis, Cardiovascular diseases, Myocardial infarction, Plaque, Coronary artery calcification, Hypertension, Intima-media thickness, Pulse wave velocity

## Abstract

**Background:**

Advanced glycation end products (AGEs) have been linked to cardiovascular disease (CVD), especially coronary heart disease (CHD), but their role in CVD pathogenesis remains unclear. Therefore, we investigated cross-sectional associations of skin AGEs with subclinical atherosclerosis, arterial stiffness, and hypertension after confirming their relation with CHD.

**Methods:**

In the population-based Rotterdam Study, skin AGEs were measured as skin autofluorescence (SAF). Prevalent MI was obtained from digital medical records. Carotid plaques, carotid intima-media thickness (IMT), coronary artery calcification (CAC), pulse wave velocity (PWV), and hypertension were assessed. Associations of SAF with endophenotypes were investigated in logistic and linear regression models adjusting for common cardiovascular risk factors. Effect modification by sex, diabetes mellitus, and chronic kidney disease (CKD) was tested.

**Results:**

3001 participants were included (mean age 73 (SD 9) years, 57% women). One unit higher SAF was associated with the presence of carotid plaques (OR 1.2 (0.92, 1.57)), a higher max IMT (0.08 SD (0.01, 0.15)), higher CAC (OR 2.2 (1.39, 3.48)), and PWV (0.09 SD (0.01, 0.16)), but not with hypertension (OR 0.99 (0.81, 1.21)). The associations with endophenotypes were more pronounced in men and participants with diabetes or CKD with significant interactions.

**Conclusions:**

Previously documented associations between SAF and CVD, also found in our study, may be explained by the endophenotypes atherosclerosis and arterial stiffness, especially in men and individuals with diabetes or CKD, but not by hypertension. Longitudinal studies are needed to replicate these findings and to test if SAF is an independent risk factor or biomarker of CVD.

*Trial registration*: The Rotterdam Study has been entered into the Netherlands National Trial Register (NTR; www.trialregister.nl) and the WHO International Clinical Trials Registry Platform (ICTRP; www.who.int/ictrp/network/primary/en/) under shared catalogue number NTR6831.

**Supplementary Information:**

The online version contains supplementary material available at 10.1186/s12933-023-02052-7.

## Background

Cardiovascular disease (CVD) is the leading cause of death worldwide [1]. Its complex pathophysiology involves lifetime metabolic disturbance and pathological alterations that usually develop asymptomatically over time. Individuals with unnoticed subclinical cardiovascular diseases are at a much higher risk of developing clinical CVD [2].

Advanced glycation end products (AGEs) have recently emerged as a novel actionable marker for diabetic cardiovascular complications [3]. They are a cluster of molecules generated irreversibly mainly on proteins through non-enzymatic glycation by reducing sugars and metabolic intermediates of lipids and sugars [3]. AGEs were suggested to contribute to the etiology of CVD by adding to tissue stiffness through forming crosslinks, and triggering inflammation through interaction with the receptor of AGEs (RAGE). They accumulate in tissues over time, especially under conditions of hyperglycemia, renal insufficiency, and smoking [4, 5]. The deposition of AGEs has been observed in the vasculature [6], atherosclerotic lesions, and cardiac tissues [7, 8] but these tissues are not easily accessible. Alternatively, skin AGEs with an estimated half-life of ~ 15 years, measured noninvasively as skin autofluorescence (SAF), are increasingly used as a proxy of AGE levels in long-lived tissues. AGEs are considered a marker of biological aging, with SAF and measures related to AGEs, i.e., the ratio between AGEs and soluble RAGE being linked to reduced survival in people who had diabetes or chronic kidney disease (CKD) [9, 10]. Moreover, SAF is regarded as a marker of long-term metabolic memory due to the close relationship of AGE formation with glucose metabolism, lipid peroxidation, and inflammation. Active metabolic management was shown to reduce the recurrence of 81.9% of cardiovascular events in young patients with premature coronary artery disease [11], calling for more attention to metabolic burden in cardiovascular health. A study in type 1 diabetes revealed that skin AGEs were associated with future intima-media thickness, and were lower in people who received intensive glucose management compared to regular management [12–14], indicating AGEs are involved in the long-term metabolic burden. In contrast, traditional CVD risk factors are likely insufficient to capture this burden.

A higher SAF level predicts independently diabetic foot ulcer that is closely linked to micro and macroangiopathies [15]. Further, it was associated with cardiovascular mortality in populations with chronic kidney disease (CKD), diabetes, or peripheral artery disease in a recent meta-analysis. SAF was related to elevated diabetes and CVD risk after a median follow-up of 4 years in a large cohort of the general population and to the incidence of major adverse cardiovascular events in patients with heart failure [16–18]. However, the associations of AGEs with cardiovascular endophenotypes remain unclear, and inconsistent findings on e.g., hypertension have been reported [19, 20]. The majority of studies focused on the relation of AGEs with a single trait in patients with diabetes or CKD, but the association of AGEs with multiple subclinical changes underlying CVD has not been fully investigated in a seemingly healthy adult population. It also remains unclear if the associations differ between men and women, diabetics and nondiabetics, and by the presence of CKD. Furthermore, many studies focused on circulating AGEs that have a much shorter half-life and are easily influenced by diet and metabolic status. Their link with CVD risk remains inconclusive while skin AGEs showed a more consistent link [21–23].

To understand the role of AGEs in the development of CVD, this study assessed how and through which subclinical cardiovascular changes skin AGEs are related to CVD in the general population and investigated potential subgroup differences in these associations. The associations of SAF with multiple endophenotypes in different vascular beds, reflecting atherosclerosis or arterial stiffness, were investigated in the well-characterized population-based Rotterdam Study after confirming an association of SAF with CVD.

## Methods

### Study population

Participants were from the population-based cohort, the Rotterdam Study (RS), which invited all inhabitants of the Ommoord suburb of Rotterdam to participate [24]. The first subcohort (RS-I) was initiated in 1990 and included 7983 participants aged 55 years and older. The second subcohort (RS-II) started with 3011 new participants aged 55 years and older in 2000. The third subcohort (RS-III) included 3932 new participants aged 45 years and older in 2006. All participants were examined at baseline and were followed up every 3–6 years at home and at the Rotterdam Study research center. The Rotterdam Study was approved by the institutional review board (Medical Ethics Committee) of Erasmus Medical Center and by the review board of The Netherlands Ministry of Health, Welfare and Sports. All participants in the present analysis provided written informed consent.

SAF was introduced to the Rotterdam Study in the middle of follow-up visits (RS-I-6th, RS-II-4th, and RS-III-2nd visits) to the RS research center from 2012 to 2016 and was measured in 3009 participants. No apparent selection bias seemed present. After excluding 8 participants with SAF outside mean ± 4SD, 3001 participants were eligible for analyses and those with available data on outcomes were included for each analysis. Population inclusion and exclusion details are shown in Additional file 1: Figure S1.

### Measurement of SAF

During visits to the RS research center, SAF at the inner side of the dominant forearm was measured using an AGE Reader (Diagnoptics B.V., Groningen, The Netherlands) based on the fluorescent property of some AGEs. It is expressed in arbitrary units (A.U.) based on the ratio between the strength of emission and excitation light. The measurement was previously validated against liquid chromatography-mass spectrometry measurements [25]. Three measurements were taken consecutively and showed good agreement (Pearson correlation coefficients 0.87–0.91). The mean was used for analyses. The device was calibrated by measuring the autofluorescence of the standard and requires re-calibration if the difference with standard autofluorescence is more than 10%. Details of the measurement have been described previously [26]. 1 A. U. corresponds to ~ 2 SD difference in SAF in our population. In healthy Dutch individuals younger than 70 years, SAF showed a slow yearly progression (~ 0.024 A. U.) and 1 A. U. difference of SAF corresponds to differences of ~ 40 years difference. However, smokers had a much higher SAF (~ 0.16 A. U.) than non-smokers [5], so as the presence of other major risk factors including diabetes and CKD. The yearly progression of SAF was also a bit faster when kidney diseases and diabetes were present [5, 27]. A quick increase of around 0.4 A. U. SAF was observed after acute renal failure [28]. SAF showed no seasonal variation in a German study and our study population [29]. To minimize the potential influence of skin creams, blood flow, and skin color, SAF was measured at the inner side of the forearm. Participants were asked not to use sunscreen and skin creams 2 days before the measurements and had tests without performing physical exercise.

### Assessment of myocardial infarction history

To confirm an association of SAF with CVD, we examined the association of SAF with a history of myocardial infarction (MI), a major type of CVD. A history of MI was defined as having had MI at study entry or had an MI event during follow-up before SAF was measured during the RS-I-6th, RS-II-4th, and RS-III-2nd visits. Data collection on MI was explained in detail elsewhere [30]. Briefly, in RS-I, the presence of MI at study entry was based on verification of self-reported MI or electrocardiogram abnormalities suggestive of previous MI. In RS-II and RS-III, the medical records of all participants were screened for prevalent MI at entry. The presence of MI was based on clinical information and ICD-10 coding from the medical records, adjudicated by two independent cardiovascular researchers. During follow-up, medical records and hospital discharge diagnoses of all participants were continuously screened for MI events.

### Assessment of subclinical endophenotypes of CVD

#### Carotid artery plaque

Both the left and right sides of the common carotid artery, carotid bifurcation, and the internal carotid artery were visualized by B-mode ultrasonography with a 7.5-MHz linear array transducer (ATL Ultra-Mark IV) over a length as large as possible. The presence of plaques at the front and rear walls of the artery was defined as focal widenings of the arterial wall ≥ 50% of adjacent segments, with protrusion into the lumen and composed of calcified or noncalcified components [31]. Participants with missing plaque information at ≥ 2 arteries were excluded. A weighted plaque score was calculated as the total number of sites (0–6) with plaque detected divided by the number of sites with an available ultrasonographic image (6 or less), and multiplied by 6 [31]. Plaque scores of (or approximately) 0, 1, 2, and at least 3, were considered indicators of no, mild, moderate, and severe plaque burden [32], respectively.

#### Carotid intima-media thickness

**Carotid intima-media thickness (IMT)** was measured using ultrasound. The max common carotid IMT was determined as the maximum of near and far wall measurements of the right (or left) carotid artery at the beginning of the dilatation of the distal common carotid artery [33].

#### Coronary artery calcification

Coronary artery calcification (CAC) was assessed using two types of computed tomography. Electron-beam tomography (EBT) was used at the RS-I-3rd visit using a C-150 scanner (Imatron, South San Francisco, California, U.S.A.) in participants not older than 85 years to scan from the level of the root of the aorta through the heart. CAC was quantified using AccuImage software (AccuImage Diagnostics Corporation, South San Francisco, California) [34]. Coronary arteries were also scanned by multi-detector computed tomography (MDCT) at the RS-I-4th and RS-II-2nd visits using 16-or 64-slice multidetector CT scanners (Somatom Sensation 16 or 64; Siemens, Forchheim, Germany) [35]. The presence and the amount of calcification were evaluated at the left main, left anterior descending, left circumflex, and right coronary arteries. CAC was quantified by the Agatston score using Syngo Calcium Scoring software (Siemens). CAC scores were categorized into four categories: 0 for the absence of calcification, 1–99 for mild calcification, 100–399 for moderate calcification, and ≥ 400 for severe calcification [36].

#### Pulse wave velocity

Pulse wave velocity (PWV) was assessed using an automatic device (Complior; Artech Medical, Pantin, France) that measures the time delay between the rapid upstroke of the feet of simultaneously recorded pulse waves in the carotid and femoral arteries. PWV was calculated by dividing the distance between the recording sites in the carotid and femoral arteries by the foot-to-foot time delay in meters/second [37].

#### Hypertension

Blood pressure was assessed at each examination round. Systolic blood pressure (SBP) and diastolic blood pressure (DBP) were measured at the right brachial artery level with the participant in sitting position. The mean of two consecutive measurements was used. Hypertension was defined as SBP ≥ 140 mmHg and/or DBP ≥ 90 mmHg or the use of blood pressure-lowering medication. The prescription of blood pressure-lowering medication for hypertension was confirmed by a physician.

The timeline of data collection is shown in Additional file 1: Figure S2.

### Assessment of covariates and population characteristics

Physical activity, smoking habits, alcohol intake, the highest level of education acquired, medications used in the past week, and medical history were collected from home interviews. Physical activity was expressed in metabolic equivalents METhours/week. Smoking status was categorized as never, past, or current smoker based on the smoking habit of cigarette, cigar, or pipe. Alcohol intake was harmonized to grams of alcohol/day. Education level was harmonized across all three RS subcohorts according to the UNESCO classification. Weight and height were measured at the research center. Fasting serum glucose, creatinine, total cholesterol, and HDL-cholesterol were measured using standard techniques. The estimated glomerular filtration rate (eGFR, mL/min per 1.73 m^2^) was calculated using the CKD-EPI equation. CKD was defined as eGFR ≤ 60 mL/min per 1.73 m^2^. Diabetes was defined as a fasting glucose of ≥ 7.0 mmol/L or a non-fasting glucose level of ≥ 11.0 mmol/L and/or the use of glucose-lowering medication, and was verified with medical records [38]. Dyslipidemia was defined as total cholesterol > 6.5 mmol/L, or use of lipid-lowering medications, or HDL < 1.0 mmol/L for men and HDL < 1.2 mmol/L for women.

### Statistical methods

Population characteristics of the total population and SAF tertile groups were summarized. To balance sex and age representation among tertile groups, we obtained tertiles of residuals of SAF regressed on age in men and women separately and created sex-specific and age adjusted SAF tertile groups. SAF as a continuous variable, z-scores of IMT, blood pressure, and PWV were used in regression analyses. CAC scores were categorized into clinically meaningful categories (< 100, and ≥ 100), and also analyzed in the natural logarithm of (CAC + 1) for analyses due to their skewed distribution and excessive zeros. The associations between SAF and endophenotypes were studied using linear regression for continuous variables or (binary or multinomial) logistic regression for categorical variables, with SAF being the independent variable regardless of the potential causal direction. Three models were constructed to adjust the associations for potential confounders that are risk factors for AGE accumulation and cardiovascular diseases. Model 1 was adjusted for age, sex, and RS subcohorts. Model 2 was additionally adjusted for cardiovascular risk factors including BMI and the presence of dyslipidemia and hypertension. To explore the influence of covariates that are potential confounders but also important determinants of SAF, we additionally adjusted for smoking, diabetes, and eGFR in model 3. Theoretically, AGEs may also act as intermediates linking risk factors such as diabetes and smoking to cardiovascular risk. The directed acyclic graph (Additional file 1: Figure S3) shows the potential relationship of included confounders with SAF and cardiovascular outcomes. Additionally, associations of conventional cardiovascular risk factors with SAF were provided. Though not the focus of this study, we also investigated the associations of continuous and categorical SAF with prevalent MI using logistic regression models. Underlying assumptions for linear and logistic regression analyses were checked, including heteroscedasticity, collinearity, nonlinear effect, and influential data points, by reviewing variation inflation factors, residual plots, and probability plots. To reduce reverse causation as much as possible, covariates measured during visits close to the assessment of outcomes and age at SAF measurement time were used for adjustment in regression models. Specifically, covariates were derived from RS-I-5, RS-II-3, and RS-III-2 visits for analyses on MI history, hypertension, IMT, and plaque presence; from RS-I-3, RS-II-1, and RS-III-1 visits for analyses on CAC and PWV.

Missing data in covariates (ranging from 0.8 to 4.1%) were imputed by multiple imputations using logistic regression and predictive mean matching methods for categorical and continuous variables respectively for 10 imputations using the MICE package in R. The results were pooled from analyses of the imputed datasets. A two-sided p < 0.05 was considered statistically significant. All analyses were conducted using R Studio (version 4.1.2).

#### Subgroup and sensitivity analysis

Because of the sex differences in cardiovascular risks and pathophysiology [39] and a likely higher accumulation of AGEs in men and people with diabetes and CKD, we examined whether sex, diabetes, or CKD modified the associations by testing their interaction terms with SAF. A two-sided p-value less than 0.1 for the interaction term was considered an indication of the presence of potential interaction and led to subsequent stratified analyses. To evaluate whether MI history drives the associations with endophenotypes, analyses were repeated after excluding participants with a history of MI.

## Results

**Fig. 1:**
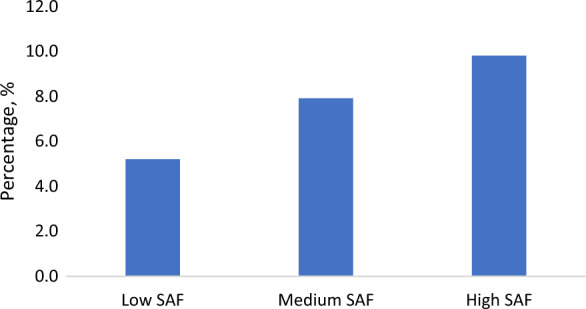
Percentage of MI history in SAF tertile groups. Percentage, percentage of individuals who had a history of MI in SAF tertile groups

A total of 3001 participants (57% women) were included in the study. They were 73 ± 9 (mean ± SD) years old, ranging from 53 to 101 years old, at the time of SAF measurement, and 229 participants (7.6%) had a history of MI (Table 1). SAF was 2.40 ± 0.49 A.U. (ranging from 1.10 to 4.40) in the total population and was higher among participants with MI history (2.64 ± 0.52) than those without (2.38 ± 0.48). Compared to participants without MI history, those who had MI were older and more often men, and they also had a higher IMT, PWV, CAC, a higher prevalence of hypertension, and a lower DBP. They also had a higher prevalence of dyslipidemia, diabetes mellitus, and chronic kidney disease, higher BMI, lower eGFR, and were more often ex-smokers(Table 1). Across SAF tertiles, there was an increasing trend in individuals who had a history of MI (Fig. 1), CAC burden, prevalence of CKD, diabetes, smokers, and use of lipid-lowering medications(Additional file 1: Table S1). Participants in the highest SAF tertile also had the highest IMT and PWV and the highest prevalence of dyslipidemia and hypertension compared to the other two groups. Regarding the comparison in subgroups stratified by sex, diabetes and CKD, SAF, IMT, CAC, PWV, and the prevalence of plaques and hypertension were higher in men than in women and higher in individuals with diabetes or CKD than in those without. Multiple other cardiovascular risk factors also differed by these strata (Additional file 1: Table S2). After adjusting for age, sex, and RS subcohorts, a lower eGFR, higher BMI, lower total cholesterol and HDL, smoking, diabetes, and dyslipidemia were all associated with higher SAF (Additional file 1: Figure S3). SBP was not associated with SAF, however, DBP was inversely associated with SAF.

**Table 1:** Characteristics of the study population, total and by the presence of prevalent myocardial infarction

	Total	Non-prevalent MI	Prevalent MI
N	3001	2767	229
Age, year	72.71 ± 9.36	72.40 ± 9.39	76.26 ± 8.31
Sex (female)	1,697 (57)	1,633 (59)	60 (26)
Caucasian	2,775 (96)	2,555 (96)	215 (97)
Smoking status			
Never	952 (32)	915 (33)	35 (16)
Former	1,619 (55)	1,460 (53)	159 (72)
Current	388 (13)	359 (13)	28 (13)
Body mass index, kg/m^2^	27.55 ± 4.30	27.49 ± 4.32	28.20 ± 4.01
Waist-hip ratio	0.90 ± 0.09	0.90 ± 0.09	0.96 ± 0.10
Total cholesterol, mmol/L	5.45 ± 1.10	5.52 ± 1.08	4.56 ± 1.01
HDL, mmol/L	1.44 (1.20, 1.74)	1.45 (1.20, 1.76)	1.26 (1.09, 1.48)
Triglycerides, mmol/L	1.27 (0.97, 1.72)	1.27 (0.98, 1.73)	1.28 (0.96, 1.72)
Lipid lowering medication	916 (31)	737 (27)	178 (80)
Dyslipidemia	1562 (54)	1364 (51)	197 (89)
eGFR, mL/min per 1.73 m^2^	74.94 (65.49, 84.46)	75.34 (65.96, 84.62)	70.16 (57.88, 81.22)
Chronic kidney disease	431 (15)	366 (14)	64 (29)
Diabetes	413 (14)	365 (13)	47 (21)
Mean intima-media thickness, mm	0.92 ± 0.16	0.92 ± 0.15	0.98 ± 0.18
Max intima-media thickness, mm	1.05 ± 0.19	1.04 ± 0.19	1.14 ± 0.23
Pulse wave velocity, m/s	11.10 (9.88, 12.62)	11.03 (9.85, 12.54)	11.86 (10.57, 13.37)
CAC score (EBT)	48.74 (3.61, 227.69)	32.50 (3.09, 182.38)	322.60 (79.30, 819.52)
Absent, mild, moderate, severe EBT-CAC scores (n)	73, 201, 101, 80	71, 190, 89, 58	2, 11, 12, 22
CAC score (MDCT)	77.40 (4.95, 363.32)	57.45 (3.18, 294.02)	541.80 (118.00, 1,397.60)
Absent, mild, moderate, severe MDCT-CAC scores (n)	52, 116, 75, 75	52, 107, 69, 56	0, 8, 6, 19
SBP, mmHg	139.08 ± 19.77	139.24 ± 19.90	137.20 ± 18.12
DBP, mmHg	76.45 ± 11.09	76.73 ± 11.09	73.12 ± 10.65
Use of blood pressure lowering medication	1,577 (53)	1,368 (50)	209 (92)
Hypertension	2,166 (72)	1,949 (70)	217 (95)
Presence of carotid plaques	2469 (85.7)	2257 (84.8)	209 (95.9)
Plaque score	2 (1, 4)	2 (1, 4)	4 (3, 5)
SAF, A.U	2.40 ± 0.49	2.38 ± 0.48	2.64 ± 0.52

*MI* myocardial infarction, *MET* the metabolic equivalent of task, with one MET defined as 1 kcal/kg/hour, *HDL* high-density lipoprotein, *eGFR* estimated glomerular filtration rate, *CAC* coronary artery calcification, *EBCT* electron-beam tomography, *MDCT* multi-detector computed tomography, *SBP* systolic blood pressure, *DBP* diastolic blood pressure, *SAF* skin autofluorescence

Percentage of missing in covariates: smoking status (1.4%), BMI (2.3%), waist-hip ratio (2.2%), total cholesterol (4.1%), HDL (4.1%), triglycerides (4.1%), lipid lowering medication (1.0%), eGFR (4.1%), chronic kidney disease (4.1%), diabetes (0.8%). Data are derived from non-imputed variables. Values are counts (valid percentages), means ± standard deviations, or medians (interquartile ranges) in the case of a skewed distribution.

### SAF and the history of myocardial infarction

A higher SAF was associated with an MI history, independent of other established cardiovascular risk factors (adjusted odds ratio of MI for one unit higher SAF 1.57 (95% CI 1.16, 2.12)) (Table 2). The highest SAF tertile had an odds ratio of 1.87 (95% CI 1.28, 2.73) compared to the lowest tertile. No interaction of SAF with sex, diabetes, and CKD was observed (p > 0.1), but the interaction of SAF and diabetes had a p-value of 0.13, which is close to 0.1. Subsequent stratified analysis showed a more pronounced association in participants who had diabetes (OR 2.83 (95% CI 1.42, 5.68)) than those without (OR 1.39 (95% CI 0.98, 1.95)) in model 3 (Additional file 1: Figure S4).

**Table 2:** Association between SAF and the history of myocardial infarction

	n/N	OR for prevalent myocardial infarction (95% CI)			
		Crude model	Model 1	Model 2	Model 3
SAF tertiles^a^					
Low SAF	52/999	1.00 (reference)	1.00 (reference)	1.00 (reference)	1.00 (reference)
Medium SAF	79/998	1.57 (1.09, 2.25)	1.72 (1.19, 2.50)	1.76 (1.20, 2.59)	1.72 (1.17, 2.54)
High SAF	98/999	1.98 (1.40, 2.81)	1.98 (1.39, 2.84)	1.93 (1.33, 2.8)	1.87 (1.28, 2.73)
One-unit increase	229/2996	2.61 (2.03, 3.35)	1.71 (1.29, 2.26)	1.63 (1.22, 2.18)	1.57 (1.16, 2.12)

n/N: number of individuals who had myocardial infarction/sample size of the group

ORs and 95% CIs are adjusted odds ratios and the respective 95% confidence intervals of myocardial infarction in association with one unit higher SAF or of the medium and high SAF groups when compared to the low SAF group

The crude model: was not adjusted for covariates

Model 1 adjusted for age, sex, and RS subcohorts

Model 2 adjusted for age, sex, RS subcohorts, body mass index, dyslipidemia, and hypertension

Model 3 adjusted for age, sex, RS subcohorts, body mass index, dyslipidemia, hypertension, smoking status, diabetes, and eGFR

^a^Sex-specific, age-adjusted SAF tertiles were calculated among n = 3001 participants

### SAF and carotid plaques

Carotid plaques were detected in 2469 of the 2882 participants with images of more than 4 carotid arteries (Table 1). Higher SAF was associated with plaque presence after adjusting for age, sex, and RS subcohorts (OR 2.24 (95% CI 1.76, 2.85)) for one unit higher SAF), but the association was attenuated in model 3 (OR 1.20 (95% CI 0.92, 1.57)) (Table 3). Plaque severity was classified as mild, moderate, and severe in 494 (17.1%), 561 (19.5%), and 1414 (49.1%) participants, respectively (Additional file 1: Table S3). Using participants who did not have plaques as the reference group, a higher SAF was only associated with the severe plaque burden (OR 1.45 (95% CI 1.09, 1.93) for one unit higher SAF) but not with the moderate (OR 0.87 (95% CI 0.64, 1.20)) and mild (OR 1.17 (95% CI 0.85, 1.60)) plaque burden in model 3. Sex modified the association of SAF with plaque presence (p for interaction = 0.03). The association of SAF with plaque presence (yes/no) was not significant in either sex in model 3, but the association with severe plaque burden appeared to be larger in men than in women. (Additional file 1: Figure S5).

**Table 3:** Associations between SAF and endophenotypes reflecting atherosclerosis or arterial stiffness

Outcome^a^	n/N	Crude model	Odds ratio (95% CI)^b^		
			Model 1	Model 2	Model 3
Hypertension	2166/2996	1.67 (1.41, 1.99)	1.11 (0.92, 1.35)	1.01 (0.83, 1.23)	0.99 (0.81, 1.21)
Plaque presence	2469/2882	2.24 (1.76, 2.85)	1.4 (1.09, 1.81)	1.36 (1.05, 1.76)	1.20 (0.92, 1.57)
Categorical CAC (EBT)	455	1.66 (1.13, 2.45)	1.31 (0.87, 1.97)	1.31 (0.87, 1.97)	1.31 (0.87, 1.97)
Categorical CAC (MDCT)	318	2.2 (1.39, 3.48)	2.2 (1.39, 3.48)	2.2 (1.39, 3.48)	2.2 (1.39, 3.48)

Outcome^a^	n/N	Crude model	Beta coefficients (95% CI)^c^		
			Model 1	Model 2	Model 3
IMT (max)	2878	0.42 (0.35, 0.5)	0.13 (0.06, 0.2)	0.11 (0.04, 0.17)	0.08 (0.01, 0.15)
log CAC (EBT)	455	0.92 (0.48, 1.35)	0.55 (0.1, 0.99)	0.39 (-0.04, 0.83)	0.28 (-0.15, 0.71)
log CAC (MDCT)	318	1.09 (0.57, 1.61)	0.77 (0.25, 1.29)	0.64 (0.14, 1.14)	0.55 (0.04, 1.06)
PWV	2402	0.41 (0.33, 0.49)	0.12 (0.04, 0.2)	0.09 (0.02, 0.17)	0.09 (0.01, 0.16)

Crude model: not adjusted for covariates

Model 1: adjusted for age, sex, and RS subcohorts

Model 2: adjusted for age, sex, RS subcohorts, body mass index, dyslipidemia, and hypertension

Model 3: adjusted for age, sex, RS subcohorts, body mass index, dyslipidemia, hypertension, smoking status, diabetes, and eGFR

For associations with hypertension, hypertension was not adjusted

*SAF* skin autofluorescence, *N* number of participants, *CI* confidence interval, *IMT* carotid intima-media thickness, *CAC* coronary artery calcification score, *EBT* electron-beam tomography, *MDCT* multi-detector computed tomography, *PWV* pulse wave velocity

^a^z-scores of IMT and PWV, categorial CAC scores (≥ 100 vs. < 100), and log-transformed (CAC + 1) were used in the analyses

^b^Odds ratios (95% CIs) were adjusted odds ratios associated with one unit higher SAF

^c^Coefficients (95% CIs) are adjusted differences of the endophenotypes associated with one unit higher SAF, in folds of SD for IMT, PWV, and the natural logarithm of (CAC + 1) for CAC

### SAF and carotid intima-media thickness

**Fig. 2:**
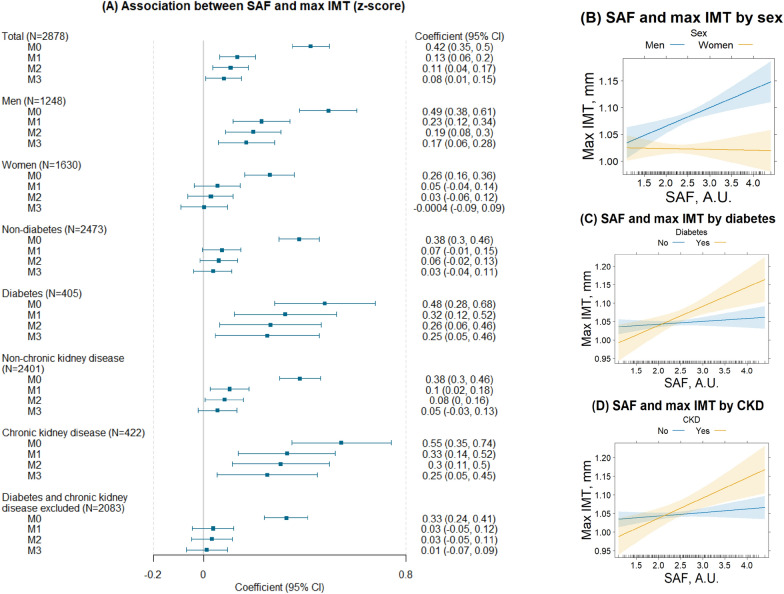
The association between SAF and max IMT by sex, and by the presence of diabetes or chronic kidney disease. Plot **A**: forest plot of coefficients and confidence intervals for the association between SAF and max IMT in the total population and subgroups in four models. M0 was the crude association not adjusted for covariates. M1 was adjusted for age, sex, and RS subcohorts. Model 2 was additionally adjusted for body mass index, dyslipidemia, and hypertension. Model 3 was additionally adjusted for smoking status, diabetes, and eGFR on top of model 2. Sex and diabetes were not adjusted in the models when stratified by sex or diabetes status. Plots **B**–**D**: effect plots of SAF by sex and the presence of diabetes or CKD. Fitted values of max IMT along SAF values in subgroups (R package “effects”). The solid line represents the predicted values of max IMT and the shade represents 95% confidence intervals with the other predictors held at the mean, deriving from the following models: **B** max IMT ~ SAF + age + sex + RS subcohorts + BMI + dyslipidemia + hypertension + smoking status + diabetes + eGFR + SAF*sex. **C** max IMT ~ SAF + age + sex + RS subcohorts + BMI + dyslipidemia + hypertension + smoking status + diabetes + eGFR + SAF*diabetes. **D** max IMT ~ SAF + age + sex + RS subcohorts + BMI + dyslipidemia + hypertension + smoking status + diabetes + eGFR + CKD + SAF*CKD

In model 3, one unit higher SAF was associated with 0.08 SD (95% CI 0.01, 0.15) higher max IMT (Table 3). Significant interactions were noticed including SAF with sex (p for interaction = 0.01), diabetes (p for interaction = 0.02), and CKD (p for interaction = 0.01). Stratified analysis showed that the association of SAF with IMT was attenuated substantially after adjusting for age and pertained in men but not women, and in individuals with either diabetes or CKD but not those without these diseases (Fig. 2). Adjusting for other risk factors further weakened the association.

### SAF and coronary artery calcification

The CAC score was measured by EBT (N = 455) or MDCT (N = 318). In model 1, one unit higher SAF was associated with a 0.55 (95% CI: 0.1, 0.99) higher EBT-CAC score in natural logarithm, and a 0.77 (95% CI: 0.25, 1.29) higher MDCT-CAC score in natural logarithm (Table 3). The associations attenuated in model 3 to 0.28 (95% CI −0.15, 0.71) for EBT-CAC and 0.55 (95% CI 0.04, 1.06) for MDCT-CAC. No significant interactions with sex, diabetes, or CKD were noticed. For categorical CAC scores, taking the category of absent or mild CAC score (CAC < 100) as the reference, a higher SAF was associated with a moderate or severe MDCT-CAC score (CAC ≥ 100), OR 2.2 (95% CI 1.39, 3.48). No significant association was observed for the categorical EBT-CAC score (OR 1.31 (95% CI 0.87, 1.97)).

### SAF and pulse wave velocity

In model 3, one unit higher SAF was associated with a 0.08 SD (95%CI 0.01, 0.15)) higher pulse wave velocity, an indicator of arterial stiffness (Table 3). A significant interaction between SAF and diabetes (p for interaction = 0.001) was observed (Additional file 1: Figure S6). A more pronounced association was noted for people who had diabetes than those who did not have diabetes, with an adjusted difference in PWV of 0.36 SD (95% CI 0.06, 0.65) vs. 0.05 SD (95% CI −0.03, 0.13) for one unit higher of SAF.

### SAF and hypertension

Hypertension was present in 2166 out of the 2996 participants with available information. No association was observed between SAF and hypertension in model 3 (OR 0.99 (95% CI 0.81, 1.21) (Table 3).

### Sensitivity analysis

The associations of SAF with all endophenotypes remained similar after excluding participants with an MI history (Additional file 1: Table S4).

## Discussion

After confirming that in a general population of middle-aged and older individuals, a higher level of skin AGEs measured as SAF was associated with prevalent MI independent of other cardiovascular risk factors, we investigated which endophenotypes of CVD might underlie this association. We found that SAF was associated with markers of subclinical atherosclerosis, including carotid plaque burden, arterial wall thickening measured by carotid IMT, and coronary artery calcification. It was also associated with arterial stiffness measured by PWV but was not associated with hypertension. The associations were independent of established cardiovascular risk factors and independent of MI history, and seemed to be more pronounced in men and in individuals who had diabetes or CKD.

The associations with endophenotypes suggest that AGEs may be related to subclinical atherosclerosis and arterial stiffness, even in people without an MI history. Our findings extend the associations from small-scale studies of mainly high-risk individuals to the general population of adequate size [19, 29, 40–42]. The association between SAF and PWV was consistent with findings in individuals of normoglycemic, prediabetic, and diabetic conditions [43]. Together with our findings, these data implicate SAF in cardiovascular risk in all glycemic strata.

Potential mechanisms underlying these associations are complex and intertwined. Carotid plaque measured by ultrasonic imaging is a surrogate outcome for CHD and indicates overall atherosclerosis burden, including the coronary artery [32]. CAC score, which measures the extent of calcification in arteries, is an indirect measure of plaque burden. Their positive association with SAF may arise because AGEs may contribute to atherosclerosis by leading to systemic vascular inflammation through activating RAGE on endothelial cells [44]. Carotid IMT can indicate the size of plaques, but the thickening of the intima-media layer can also result from age-related vascular remodeling that involves AGE-mediated crosslinks on elastin and collagen [45]. These crosslinks disturb the normal structure of the arterial wall and add to arterial stiffness [46], also contributing to the positive association between SAF and PWV. Further, AGEs were reported to disturb multiple cellular activities in the progression of atherosclerosis and arterial stiffness, such as lipids transportation [47], endothelial nitric oxide production [48], and HDL antioxidative capacity [49].

### AGEs and blood pressure

Given a likely role of AGEs in arterial stiffness, an association of a higher SAF with hypertension would be expected. However, the current study fuels the debate of whether AGEs are involved in hypertension by showing no association [50]. DBP was even inversely associated with SAF, which was also observed in a younger Dutch cohort NQplus in men but not in women [51]. It is widely perceived that DBP tends to decrease after around 50–60 years of age mainly due to stiffness of large arteries [52]. As such, our observations may imply that AGEs contribute to large artery stiffness by forming crosslinks on elastin or collagen during the aging process. The results may not be extrapolated to younger populations because the majority of our study participants were at the later part of the blood pressure trajectory where DBP tends to decrease and the increase of SBP with age slows down [53]. In fact, the Dutch LifeLines cohort covering a wider age range (n = 78671) reported that higher SAF was associated with hypertension in men [54]. Nevertheless, a null association between SAF and BP was also observed in primary school children [55]. We encourage longitudinal studies to investigate the link between AGEs and hemodynamic patterns to address their relevance to cardiovascular risk.

### Subgroup differences of the associations between AGEs and IMT

The association of SAF with IMT showed subgroup differences. It was stronger in people who had diabetes or CKD and was insignificant in those without and was seen in men but not women, arguing for a more pronounced impact of AGEs on IMT in diabetes or CKD, and in men.

Diabetes and CKD are two major risk factors for AGE accumulation and CVD. Our observation may indicate that AGE accumulation is involved in arterial wall thickening and further contributes to an elevated CVD risk in diabetes or CKD. In line with our finding, a study in people with type 1 diabetes showed that, compared to regular glucose management, 6.5 years of intensive glucose management resulted in a lower IMT [13] 6 years after the intervention and the levels of skin AGEs and IMT correlated [12]. Likewise, we observed a larger association between SAF and PWV in people with diabetes than in those without but the large time gap between measurements limits the interpretation of results.

Sex differences in the association between SAF and IMT suggest that SAF is more relevant for IMT in men. This difference may be attributed to the better risk profile of women. Moreover, women generally have a lower IMT than men before menopause potentially due to a lower grade of chronic inflammation [56], protection from estrogen [57], or a better risk profile [58]. The sex disparity diminishes gradually afterward [59]. Although all participants in our study were postmenopausal, the long half-life of skin AGEs (median 15 years) may capture associations of skin AGEs and IMT from earlier life stages, i.e., a potential absence of association in women before menopause. Similarly, we observed an association of SAF with severe plaque burden only in men. Future longitudinal studies could investigate whether these differences translate to sex disparities in CVD risk [39]. Our results shed light on how subclinical cardiovascular pathological changes in relation to SAF might develop differently in men and women and individuals with or without diabetes or CKD.

### Strengths and limitations

Strengths of our study include the analysis in a well-characterized and densely phenotyped general population with well-defined outcomes, allowing for investigating multiple CVD-related endophenotypes within one general population of adequate sample size and controlling for multiple confounders. SAF, as a marker for long-term AGEs accumulation, can capture the accumulated AGE burden in tissues during a longer period than circulating markers.

Several limitations are also present in the current study. Our results were restricted to the older Dutch population and the generalization of results needs caution. The small group of participants with diabetes or CKD and CAC data also limits the power for statistical inference. Further, we were not able to rule out influences from survival selection and were only able to investigate the association of SAF with a history of non-fatal MI as the clinical outcome of fatal MI before SAF measurement was automatically precluded. Nevertheless, in both cases, selection would likely have biased the associations toward the null. In addition, reverse causation might have biased the estimations because some outcomes were assessed before SAF measurement although SAF showed a slow yearly progression [5]. For this reason, we repeated our analyses in participants without a history of MI as a sensitivity analysis. In addition, progression in endophenotypes measured earlier was not accounted for, which may have concealed some of the associations. Studies in CKD patients showed that one year increase in SAF was predictive of cardiovascular events and all-cause mortality [27, 60]. Future studies with repeated measurement of SAF and longitudinal data on CVD incidence may investigate if changes in SAF are related to cardiovascular risk in the general population. Although we were able to control for many risk factors, given the intertwined relationship of SAF with many risk factors, residual confounding could not be ruled out completely. Finally, the cross-sectional design does not allow for the interpretation of causality.

## Conclusions

To conclude, we observed associations between skin AGEs and the extent of several endophenotypes of CVD, suggesting that AGEs may contribute to CVD by being involved in processes like atherosclerosis and arterial stiffness but not hypertension in this general population of older adults. The study also provides more data on the inconclusive association between SAF and hypertension and suggests that inconsistent observations may arise from varied age ranges of study populations. As a marker of metabolic memory, a higher SAF may signal vascular aging and subclinical cardiovascular changes beyond traditional risk factors, especially in men and individuals with diabetes or CKD. Future risk modeling studies may investigate whether and in which groups of the population measuring SAF will benefit CVD risk prediction in clinical practice. As AGEs accumulate even in children, life-course management of AGEs may benefit cardiovascular health. Prospective studies using a longitudinal design are warranted to confirm our cross-sectional findings and evaluate if AGEs are also involved in pathological changes at later stages of CVD.

### Supplementary Information

**Additional file 1: Table S1.** Characteristics of the study population by sex- and age-adjusted SAF tertiles. **Table S2.** Characteristics of the study population by sex, and the presence of diabetes, or chronic kidney disease. **Table S3.** Association between SAF and the severity of carotid plaques in the total population. **Table S4.** Associations between SAF and endophenotypes reflecting atherosclerosis or arterial stiffness after excluding individuals with a history of myocardial infarction. **Figure S1.** Participants inclusion and exclusion flowchart and analysis scheme. **Figure S2.** Timeline of data collection. **Figure S3.** The directed acyclic graph for confounder selection. **Figure S4.** Forest plot for the association between conventional cardiovascular risk factors and SAF. **Figure S5.** The association between SAF and myocardial infarction history by the presence of diabetes or not. **Figure S6.** The association between SAF and carotid plaques in men and women. **Figure S7.** The association between SAF and pulse wave velocity by the presence of diabetes or not.

## Data Availability

The datasets generated and/or analysed during the current study are not publicly available due to restrictions based on privacy regulations and informed consent of the participants but are available from the management team of the Rotterdam Study (secretariat.epi@erasmusmc.nl) on reasonable request.

## Abbreviations

AGEs: Advanced glycation end products
CVD: Cardiovascular disease
MI: Myocardial infarction
SAF: Skin autofluorescence
IMT: Intima-media thickness
CAC: Coronary artery calcification
PWV: Pulse wave velocity
SD: Standard deviation
RAGE: Receptor for AGEs
SBP: Systolic blood pressure
DBP: Diastolic blood pressure
BMI: Body mass index
HDL: High-density lipoproteins
eGFR: Estimated glomerular filtration rate
CKD: Chronic kidney disease

## References

[1] Collaborators GCD (2018). GBD 2017 Causes of Death Collaborators. Global, regional, and national age-sex-specific mortality for 282 causes of death in;195 countries and territories, 1980–2017: a systematic analysis for the Global Burden of Disease Study 2017 (vol 392, pg 1736, 2018). Lancet.

[2] Fuchs A, Kuhl JT, Sigvardsen PE, Afzal S, Knudsen AD, Moller MB (2023). Subclinical coronary atherosclerosis and risk for myocardial infarction in a danish cohort : a prospective observational cohort study. Ann Intern Med.

[3] Singh R, Barden A, Mori T, Beilin L (2001). Advanced glycation end-products: a review. Diabetologia.

[4] Rajaobelina K, Helmer C, Velayoudom-Cephise FL, Nov S, Farges B, Pupier E (2017). Progression of skin autofluorescence of AGEs over 4 years in patients with type 1 diabetes. Diabetes Metab Res Rev.

[5] Koetsier M, Lutgers HL, de Jonge C, Links TP, Smit AJ, Graaff R (2010). Reference values of skin autofluorescence. Diabetes Technol The.

[6] Baumann M, Richart T, Sollinger D, Pelisek J, Roos M, Kouznetsova T (2009). Association between carotid diameter and the advanced glycation end product N-epsilon-carboxymethyllysine (CML). Cardiovasc Diabetol.

[7] Sakata N, Imanaga Y, Meng J, Tachikawa Y, Takebayashi S, Nagai R (1998). Immunohistochemical localization of different epitopes of advanced glycation end products in human atherosclerotic lesions. Atherosclerosis.

[8] Nakamura Y, Horii Y, Nishino T, Shiiki H, Sakaguchi Y, Kagoshima T (1993). Immunohistochemical localization of advanced glycosylation end products in coronary atheroma and cardiac tissue in diabetes mellitus. Am J Pathol.

[9] Sabbatinelli J, Castiglione S, Macri F, Giuliani A, Ramini D, Vinci MC (2022). Circulating levels of AGEs and soluble RAGE isoforms are associated with all-cause mortality and development of cardiovascular complications in type 2 diabetes: a retrospective cohort study. Cardiovasc Diabetol.

[10] Majchrzak C, Cougnard-Gregoire A, Le-Goff M, Feart C, Delcourt C, Reydit M (2022). Skin autofluorescence of advanced glycation end-products and mortality in older adults: the roles of chronic kidney disease and diabetes. Nutr Metab Cardiovasc Dis.

[11] Martinez-Sanchez FD, Medina-Urrutia AX, Jorge-Galarza E, Martinez-Alvarado MDR, Reyes-Barrera J, Osorio-Alonso H (2022). Effect of metabolic control on recurrent major adverse cardiovascular events and cardiovascular mortality in patients with premature coronary artery disease: results of the Genetics of Atherosclerotic Disease study. Nutr Metab Cardiovasc Dis.

[12] Monnier VM, Sun W, Gao X, Sell DR, Cleary PA, Lachin JM (2015). Skin collagen advanced glycation endproducts (AGEs) and the long-term progression of sub-clinical cardiovascular disease in type 1 diabetes. Cardiovasc Diabetol.

[13] Nathan DM, Lachin J, Cleary P, Orchard T, Brillon DJ, Backlund JY (2003). Intensive diabetes therapy and carotid intima-media thickness in type 1 diabetes mellitus. N Engl J Med.

[14] Monnier VM, Bautista O, Kenny D, Sell DR, Fogarty J, Dahms W (1999). Skin collagen glycation, glycoxidation, and crosslinking are lower in subjects with long-term intensive versus conventional therapy of type 1 diabetes: relevance of glycated collagen products versus HbA1c as markers of diabetic complications. DCCT skin collagen ancillary study group. diabetes control and complications trial. Diabetes.

[15] Borderie G, Foussard N, Larroumet A, Blanco L, Barbet-Massin MA, Ducos C (2023). The skin autofluorescence of advanced glycation end-products relates to the development of foot ulcers in type 2 diabetes: a longitudinal observational study. J Diabetes Complicat.

[16] Cavero-Redondo I, Soriano-Cano A, Alvarez-Bueno C, Cunha PG, Martinez-Hortelano JA, Garrido-Miguel M (2018). Skin autofluorescence-indicated advanced glycation end products as predictors of cardiovascular and all-cause mortality in high-risk subjects: a systematic review and meta-analysis. J Am Heart Assoc.

[17] van Waateringe RP, Fokkens BT, Slagter SN, van der Klauw MM, van Vliet-Ostaptchouk JV, Graaff R (2019). Skin autofluorescence predicts incident type 2 diabetes, cardiovascular disease and mortality in the general population. Diabetologia.

[18] Kunimoto M, Yokoyama M, Shimada K, Matsubara T, Aikawa T, Ouchi S (2021). Relationship between skin autofluorescence levels and clinical events in patients with heart failure undergoing cardiac rehabilitation. Cardiovasc Diabetol.

[19] Yoshioka K (2018). Skin autofluorescence is a noninvasive surrogate marker for diabetic microvascular complications and carotid intima-media thickness in japanese patients with type 2 diabetes: a cross-sectional study. Diabetes Ther.

[20] Watfa G, Soulis G, Tartagni E, Kearney-Schwartz A, Borghi C, Salvi P (2012). Relationship between tissue glycation measured by autofluorescence and pulse wave velocity in young and elderly non-diabetic populations. Diabetes Metab.

[21] Hanssen N, Engelen L, Ferreira I, Scheijen J, Huijberts M, van Greevenbroek M (2012). Plasma advanced glycation end products are not associated with cardiovascular disease in individuals with or without type 2 diabetes: the Hoorn and CODAM studies. Diabetologia.

[22] Gelzinsky J, Mayer O, Seidlerova J, Materankova M, Mares S, Kordikova V (2021). Serum biomarkers, skin autofluorescence and other methods. Which parameter better illustrates the relationship between advanced glycation end products and arterial stiffness in the general population?. Hypertens Res.

[23] Kilhovd BK, Juutilainen A, Lehto S, Ronnemaa T, Torjesen PA, Birkeland KI (2005). High serum levels of advanced glycation end products predict increased coronary heart disease mortality in nondiabetic women but not in nondiabetic men—a population-based 18-year follow-up study. Arterioscl Throm Vas.

[24] Ikram MA, Brusselle G, Ghanbari M, Goedegebure A, Ikram MK, Kavousi M (2020). Objectives, design and main findings until 2020 from the Rotterdam study. Eur J Epidemiol.

[25] Meerwaldt R, Links T, Graaff R, Thorpe SR, Baynes JW, Hartog J (2005). Simple noninvasive measurement of skin autofluorescence. Ann Ny Acad Sci.

[26] Chen J, van der Duin D, Campos-Obando N, Ikram MA, Nijsten TEC, Uitterlinden AG (2019). Serum 25-hydroxyvitamin D(3) is associated with advanced glycation end products (AGEs) measured as skin autofluorescence: the Rotterdam study. Eur J Epidemiol.

[27] Arsov S, Trajceska L, van Oeveren W, Smit AJ, Dzekova P, Stegmayr B (2013). Increase in skin autofluorescence and release of heart-type fatty acid binding protein in plasma predicts mortality of hemodialysis patients. Artif Organs.

[28] Lavielle A, Rubin S, Boyer A, Moreau K, Rajaobelina K, Combe C (2017). Skin autofluorescence in acute kidney injury. Crit Care.

[29] Du T, Brandl B, Hauner H, Skurk T. Skin autofluorescence mirrors surrogate parameters of vascular aging: an enable study. Nutrients. 2023;15(7):1597. 10.3390/nu15071597. 10.3390/nu15071597 PMC10096848 37049440

[30] Leening MJ, Kavousi M, Heeringa J, van Rooij FJ, Verkroost-van Heemst J, Deckers JW (2012). Methods of data collection and definitions of cardiac outcomes in the Rotterdam study. Eur J Epidemiol.

[31] Hollander M, Bots ML, Del Sol AI, Koudstaal PJ, Witteman JC, Grobbee DE (2002). Carotid plaques increase the risk of stroke and subtypes of cerebral infarction in asymptomatic elderly: the Rotterdam study. Circulation.

[32] van der Meer IM, Bots ML, Hofman A, del Sol AI, van der Kuip DA, Witteman JC (2004). Predictive value of noninvasive measures of atherosclerosis for incident myocardial infarction: the Rotterdam study. Circulation.

[33] Bots ML, Hoes AW, Koudstaal PJ, Hofman A, Grobbee DE (1997). Common carotid intima-media thickness and risk of stroke and myocardial infarction—the Rotterdam study. Circulation.

[34] Vliegenthart R, Oudkerk M, Song B, van der Kuip DAM, Hofman A, Witteman JCM (2002). Coronary calcification detected by electron-beam computed tomography and myocardial infarction—the Rotterdam coronary calcification study. Eur Heart J.

[35] Bos D, Leening MJG, Kavousi M, Hofman A, Franco OH, van der Lugt A (2015). Comparison of atherosclerotic calcification in major vessel beds on the risk of all-cause and cause-specific mortality the Rotterdam study. Circ-Cardiovasc Imag.

[36] Greenland P, Bonow RO, Brundage BH, Budoff MJ, Eisenberg MJ, Grundy SM (2007). ACCF/AHA 2007 clinical expert consensus document on coronary artery calcium scoring by computed tomography in global cardiovascular risk assessment and in evaluation of patients with chest pain: a report of the American College of Cardiology Foundation Clinical Expert Consensus Task Force (ACCF/AHA Writing Committee to Update the 2000 Expert Consensus Document on Electron Beam Computed Tomography) developed in collaboration with the Society of Atherosclerosis Imaging and Prevention and the Society of Cardiovascular Computed Tomography. J Am Coll Cardiol.

[37] Mattace-Raso FUS, van der Cammen TJM, Hofman A, van Popele NM, Bos ML, Schalekamp MADH (2006). Arterial stiffness and risk of coronary heart disease and stroke - The Rotterdam Study. Circulation.

[38] van Herpt TTW, Ligthart S, Leening MJG, van Hoek M, Lieverse AG, Ikram MA (2020). Lifetime risk to progress from pre-diabetes to type 2 diabetes among women and men: comparison between American Diabetes Association and World Health Organization diagnostic criteria. BMJ Open Diabetes Res Care.

[39] Leening MJ, Ferket BS, Steyerberg EW, Kavousi M, Deckers JW, Nieboer D (2014). Sex differences in lifetime risk and first manifestation of cardiovascular disease: prospective population based cohort study. BMJ.

[40] Hangai M, Takebe N, Honma H, Sasaki A, Chida A, Nakano R (2016). Association of advanced glycation end products with coronary artery calcification in Japanese subjects with type 2 diabetes as assessed by skin autofluorescence. J Atheroscler Thromb.

[41] Monnier VM, Sun WJ, Gao XY, Sell DR, Cleary PA, Lachin JM (2015). Skin collagen advanced glycation endproducts (AGEs) and the long-term progression of sub-clinical cardiovascular disease in type 1 diabetes. Cardiovasc Diabetol.

[42] Saz-Lara A, Alvarez-Bueno C, Martinez-Vizcaino V, Notario-Pacheco B, Sequi-Dominguez I, Cavero-Redondo I (2020). Are advanced glycation end products in skin associated with vascular dysfunction markers? A meta-analysis. Int J Environ Res Public Health.

[43] Birukov A, Cuadrat R, Polemiti E, Eichelmann F, Schulze MB (2021). Advanced glycation end-products, measured as skin autofluorescence, associate with vascular stiffness in diabetic, pre-diabetic and normoglycemic individuals: a cross-sectional study. Cardiovasc Diabetol.

[44] Harja E, Bu DX, Hudson BI, Chang JS, Shen X, Hallam K (2008). Vascular and inflammatory stresses mediate atherosclerosis via RAGE and its ligands in apoE-/- mice. J Clin Invest.

[45] Stirban A, Gawlowski T, Roden M (2014). Vascular effects of advanced glycation endproducts: clinical effects and molecular mechanisms. Mol Metab.

[46] Brüel A, Oxlund H (1996). Changes in biomechanical properties, composition of collagen and elastin, and advanced glycation endproducts of the rat aorta in relation to age. Atherosclerosis.

[47] Rabbani N, Godfrey L, Xue MZ, Shaheen F, Geoffrion M, Milne R (2011). Glycation of LDL by methylglyoxal increases arterial atherogenicity a possible contributor to increased risk of cardiovascular disease in diabetes. Diabetes.

[48] Ren XM, Ren LQ, Wei Q, Shao H, Chen L, Liu NF (2017). Advanced glycation end-products decreases expression of endothelial nitric oxide synthase through oxidative stress in human coronary artery endothelial cells. Cardiovasc Diabetol.

[49] Mulder DJ, De Boer JF, Graaff R, De Vries R, Annema W, Lefrandt JD (2011). Skin autofluorescence is inversely related to hdl anti-oxidative capacity in type 2 diabetes mellitus. Atherosclerosis Supp.

[50] Baumann M (2012). Role of advanced glycation end products in hypertension and cardiovascular risk: human studies. J Am Soc Hypertens.

[51] Botros N, Sluik D, van Waateringe RP, de Vries JHM, Geelen A, Feskens EJM (2017). Advanced glycation end-products (AGEs) and associations with cardio-metabolic, lifestyle, and dietary factors in a general population: the NQplus study. Diabetes Metab Res Rev.

[52] Franklin SS, Wt Gustin, Wong ND, Larson MG, Weber MA, Kannel WB (1997). Hemodynamic patterns of age-related changes in blood pressure. The Framingham Heart study. Circulation.

[53] Portegies ML, Mirza SS, Verlinden VJ, Hofman A, Koudstaal PJ, Swanson SA (2016). Mid- to Late-Life trajectories of blood pressure and the risk of stroke: the Rotterdam study. Hypertension.

[54] van Waateringe RP, Slagter SN, van Beek AP, van der Klauw MM, van Vliet-Ostaptchouk JV, Graaff R (2017). Skin autofluorescence, a non-invasive biomarker for advanced glycation end products, is associated with the metabolic syndrome and its individual components. Diabetol Metab Syndr.

[55] Kochli S, Endes K, Trinkler M, Mondoux M, Zahner L, Hanssen H (2020). Association of physical fitness with skin autofluorescence-derived advanced glycation end products in children. Pediatr Res.

[56] Gubbels Bupp MR (2015). Sex, the aging immune system, and chronic disease. Cell Immunol.

[57] Naessen T, Rodriguez-Macias K (2006). Menopausal estrogen therapy counteracts normal aging effects on intima thickness, media thickness and intima/media ratio in carotid and femoral arteries—an investigation using noninvasive high-frequency ultrasound. Atherosclerosis.

[58] Juonala M, Kahonen M, Laitinen T, Hutri-Kahonen N, Jokinen E, Taittonen L (2008). Effect of age and sex on carotid intima-media thickness, elasticity and brachial endothelial function in healthy adults: the cardiovascular risk in Young Finns Study. Eur Heart J.

[59] Sinning C, Wild PS, Echevarria FM, Wilde S, Schnabel R, Lubos E (2011). Sex differences in early carotid atherosclerosis (from the community-based Gutenberg-Heart Study). Am J Cardiol.

[60] Shardlow A, McIntyre NJ, Kolhe NV, Nellums LB, Fluck RJ, McIntyre CW (2020). The association of skin autofluorescence with cardiovascular events and all-cause mortality in persons with chronic kidney disease stage 3: A prospective cohort study. Plos Med.

