# Supplementary material for: Advanced glycation end products measured by skin autofluorescence and subclinical cardiovascular disease: the Rotterdam Study
Source: Cardiovasc Diabetol. 2023 Nov 28;22:326. doi: 10.1186/s12933-023-02052-7 (PMC10685533; doi:10.1186/s12933-023-02052-7)
Supplement: Supplementary file 1 — Additional file 1: Table S1. Characteristics of the study population by sex- and age-adjusted SAF tertiles. Table S2. Characteristics of the study population by sex, and the presence of diabetes, or chronic kidney disease. Table S3. Association between SAF and the severity of carotid plaques in the total population. Table S4. Associations between SAF and endophenotypes reflecting atherosclerosis or arterial stiffness after excluding individuals with a history of myocardial infarction. Figure S1. Participants inclusion and exclusion flowchart and analysis scheme. Figure S2. Timeline of data collection. Figure S3. The directed acyclic graph for confounder selection. Figure S4. Forest plot for the association between conventional cardiovascular risk factors and SAF. Figure S5. The association between SAF and myocardial infarction history by the presence of diabetes or not. Figure S6. The association between SAF and carotid plaques in men and women. Figure S7. The association between SAF and pulse wave velocity by the presence of diabetes or not. [file 12933_2023_2052_MOESM1_ESM.docx]

Table of Contents

[Table S1 Characteristics of the study population by sex- and age-adjusted SAF tertiles 2](#_Toc148423639)

[Table S2 Characteristics of the study population by sex, and the presence of diabetes, or chronic kidney disease 3](#_Toc148423640)

[Table S3 Association between SAF and the severity of carotid plaques in the total population 5](#_Toc148423641)

[Table S4 Associations between SAF and endophenotypes reflecting atherosclerosis or arterial stiffness after excluding individuals with a history of myocardial infarction 6](#_Toc148423642)

[Figure S1 Participants inclusion and exclusion flowchart and analysis scheme 7](#_Toc148423643)

[Figure S2 Timeline of data collection 8](#_Toc148423644)

[Figure S3 The directed acyclic graph for confounder selection 9](#_Toc148423645)

[Figure S4 Forest plot for the association between conventional cardiovascular risk factors and SAF 10](#_Toc148423646)

[Figure S5 The association between SAF and myocardial infarction history by the presence of diabetes or not 11](#_Toc148423647)

[Figure S6 The association between SAF and carotid plaques in men and women 12](#_Toc148423648)

[Figure S7 The association between SAF and pulse wave velocity by the presence of diabetes or not 13](#_Toc148423649)

**Advanced glycation end products measured by skin autofluorescence and coronary heart disease: The Rotterdam Study**

Jinluan Chen, Banafsheh Arshi, Komal Waqas, Tianqi Lu, Daniel Bos, M. Arfan Ikram, André G. Uitterlinden, Maryam Kavousi, M. Carola Zillikens

**Table S1 Characteristics of the study population by sex- and age-adjusted SAF tertiles**

|  | **Total** | **Low SAF** | **Medium SAF** | **High SAF** |
| --- | --- | --- | --- | --- |
| **N** | 3001 | 999 | 1001 | 1001 |
| **Age,** year | 72.71 ± 9.36 | 73.07 ± 9.46 | 71.59 ± 9.48 | 73.46 ± 9.04 |
| **Sex** (female) | 1,697 (57) | 565 (57) | 566 (57) | 566 (57) |
| **Caucasian** | 2,775 (96) | 921 (94) | 924 (96) | 930 (97) |
| **Smoking status** |  |  |  |  |
| Never | 952 (32) | 361 (37) | 307 (31) | 284 (29) |
| Former | 1,619 (55) | 542 (55) | 561 (57) | 516 (52) |
| Current | 388 (13) | 86 (9) | 119 (12) | 183 (19) |
| **Body mass index,** kg/m^2^ | 27.55 ± 4.30 | 27.22 ± 3.94 | 27.48 ± 4.27 | 27.93 ± 4.64 |
| **Waist-hip ratio** | 0.90 ± 0.09 | 0.90 ± 0.09 | 0.90 ± 0.10 | 0.91 ± 0.10 |
| **Total cholesterol,** mmol/L | 5.45 ± 1.10 | 5.51 ± 1.11 | 5.51 ± 1.09 | 5.33 ± 1.10 |
| **HDL,** mmol/L | 1.44 (1.20, 1.74) | 1.46 (1.21, 1.78) | 1.44 (1.21, 1.76) | 1.41 (1.16, 1.69) |
| **Triglycerides,** mmol/L | 1.27 (0.97, 1.72) | 1.26 (0.97, 1.70) | 1.25 (0.97, 1.67) | 1.31 (0.99, 1.77) |
| **Lipid lowering medication** | 916 (31) | 287 (29) | 294 (30) | 335 (34) |
| **Dyslipidemia** | 1562 (54) | 511 (52) | 508 (52) | 543 (57) |
| **eGFR,** mL/min per 1.73 m^2^ | 74.94 (65.49, 84.46) | 75.50 (66.46, 84.33) | 74.96 (65.93, 84.83) | 74.56 (63.56, 84.29) |
| **Chronic kidney disease** | 431 (15) | 126 (13) | 132 (14) | 173 (18) |
| **Diabetes** | 413 (14) | 105 (11) | 120 (12) | 188 (19) |
| **Mean intima-media thickness,** mm | 0.92 ± 0.16 | 0.92 ± 0.15 | 0.91 ± 0.15 | 0.93 ± 0.16 |
| **Max intima-media thickness,** mm | 1.05 ± 0.19 | 1.04 ± 0.19 | 1.03 ± 0.18 | 1.07 ± 0.21 |
| **Pulse wave velocity,** m/s | 11.10 (9.88, 12.62) | 11.02 (9.92, 12.51) | 10.87 (9.75, 12.41) | 11.40 (10.06, 12.90) |
| **CAC score (EBT)** | 48.74 (3.61, 227.69) | 33.65 (2.06, 194.63) | 34.94 (2.12, 210.61) | 71.17 (8.51, 279.14) |
| **CAC score (MDCT)** | 77.40 (4.95, 363.32) | 53.65 (3.88, 289.82) | 62.30 (3.60, 363.40) | 141.80 (12.30, 446.75) |
| **SBP,** mmHg | 139.08 ± 19.77 | 139.87 ± 19.70 | 138.85 ± 19.74 | 138.53 ± 19.88 |
| **DBP,** mmHg | 76.45 ± 11.09 | 77.10 ± 11.10 | 77.24 ± 11.04 | 75.03 ± 11.00 |
| **Blood pressure lowering medication** | 1,577 (53) | 513 (52) | 486 (49) | 578 (58) |
| **Hypertension** | 2,166 (72) | 715 (72) | 706 (71) | 745 (75) |
| **Presence of carotid plaques** | 2469 (86) | 815 (84) | 814 (84) | 840 (88) |
| **Plaque score** | 2 (1, 4) | 2 (1, 4) | 2 (1, 4) | 3 (1, 4) |
| **Myocardial infarction** | 229 (8) | 52 (5) | 79 (8) | 98 (10) |
| **SAF,** A.U. | 2.40 ± 0.49 | 1.94 ± 0.23 | 2.34 ± 0.20 | 2.92 ± 0.37 |

Note:

Abbreviations: HDL, High-density lipoprotein; SBP, Systolic blood pressure; DBP, Diastolic blood pressure; CAC, Coronary artery calcification; EBT, electron-beam tomography; MDCT, multi-detector computed tomography; CHD, coronary heart disease.

Data are from non-imputed variables. Values are counts (valid percentages), means ± standard deviations or median (interquartile range) in case of a skewed distribution.

**Table S2 Characteristics of the study population by sex, and the presence of diabetes, or chronic kidney disease**

|  | **Men (N = 1304)** | **Women**  **(N = 1697)** | **Non-diabetes**  **(N = 2563)** | **Diabetes (N = 413)** | **Non-CKD (N = 2448)** | **CKD (N = 431)** |
| --- | --- | --- | --- | --- | --- | --- |
| Age, year | 72.51 ± 9.31 | 72.86 ± 9.41 | 72.33 ± 9.50 | 74.80 ± 8.32 | 71.76 ± 9.19 | 77.47 ± 8.80 |
| Smoking status   Never | 261 (20) | 691 (41) | 833 (33) | 119 (29) | 805 (33) | 120 (28) |
| Former | 818 (64) | 801 (48) | 1,373 (54) | 246 (60) | 1,310 (54) | 257 (60) |
| Current | 205 (16) | 183 (11) | 342 (13) | 46 (11) | 321 (13) | 51 (12) |
| Body mass index, kg/m2 | 27.42 ± 3.61 | 27.64 ± 4.76 | 27.24 ± 4.18 | 29.45 ± 4.52 | 27.45 ± 4.29 | 27.95 ± 4.15 |
| Total cholesterol, mmol/L | 5.10 ± 1.08 | 5.72 ± 1.04 | 5.57 ± 1.07 | 4.70 ± 1.01 | 5.48 ± 1.09 | 5.27 ± 1.15 |
| HDL cholesterol, mmol/L | 1.28 (1.08, 1.51) | 1.58 (1.33, 1.91) | 1.47 (1.22, 1.78) | 1.27 (1.08, 1.53) | 1.45 (1.21, 1.76) | 1.33 (1.13, 1.64) |
| Triglycerides, mmol/L | 1.30 (0.98, 1.75) | 1.26 (0.97, 1.71) | 1.25 (0.96, 1.69) | 1.41 (1.06, 1.93) | 1.26 (0.97, 1.70) | 1.35 (1.04, 1.79) |
| Lipid lowering medication | 476 (37) | 440 (26) | 660 (26) | 256 (62) | 717 (29) | 170 (39) |
| Dyslipidemia | 680 (54) | 882 (54) | 1260 (50) | 302 (75) | 1267 (52) | 266 (62) |
| eGFR, mL/min per 1.73 m^2^ | 74.69 (65.58, 84.35) | 75.47 (65.37, 84.54) | 74.90 (65.73, 84.26) | 75.87 (63.85, 85.50) | 77.85 (70.05, 85.77) | 53.67 (47.95, 57.18) |
| Chronic kidney disease | 195 (16) | 236 (15) | 357 (14) | 74 (19) | 0 (0) | 431 (100) |
| Diabetes | 207 (16) | 206 (12) | 0 (0) | 413 (100) | 321 (13) | 74 (17) |
| Intima-media thickness (mean), mm | 0.95 ± 0.16 | 0.90 ± 0.15 | 0.91 ± 0.15 | 0.96 ± 0.17 | 0.91 ± 0.15 | 0.97 ± 0.16 |
| Intima-media thickness (max), mm | 1.09 ± 0.20 | 1.02 ± 0.18 | 1.04 ± 0.19 | 1.11 ± 0.21 | 1.04 ± 0.19 | 1.11 ± 0.21 |
| Pulse wave velocity, m/s | 11.51 (10.27, 13.20) | 10.74 (9.61, 12.18) | 10.94 (9.78, 12.41) | 12.23 (10.77, 13.91) | 10.95 (9.79, 12.40) | 12.08 (10.45, 13.59) |
| CAC score (EBT) | 108.56 (11.35, 445.60) | 17.15 (1.35, 144.22) | 3.75 (1.41, 5.36) | 4.36 (2.08, 5.98) | 33.78 (2.45, 184.25) | 84.97 (10.83, 374.18) |
| CAC score (MDCT) | 143.00 (34.10, 610.10) | 34.60 (0.90, 206.80) | 4.32 (1.50, 5.82) | 4.68 (2.97, 6.09) | 58.30 (3.20, 310.00) | 170.10 (47.10, 556.30) |
| Systolic blood pressure, mmHg | 138.21 ± 18.17 | 139.76 ± 20.90 | 138.44 ± 19.72 | 142.21 ± 19.13 | 138.42 ± 19.42 | 141.98 ± 20.86 |
| Diastolic blood pressure, mmHg | 76.93 ± 11.06 | 76.08 ± 11.11 | 76.71 ± 11.09 | 74.91 ± 10.94 | 76.62 ± 11.04 | 75.34 ± 11.17 |
| Blood pressure lowering medication | 711 (55) | 866 (52) | 1,237 (49) | 326 (80) | 1,204 (50) | 298 (70) |
|  | **Men (N = 1304)** | **Women**  **(N = 1697)** | **Non-diabetes**  **(N = 2563)** | **Diabetes (N = 413)** | **Non-CKD (N = 2448)** | **CKD (N = 431)** |
| Hypertension | 967 (74) | 1,199 (71) | 1,782 (70) | 367 (89) | 1,698 (69) | 374 (87) |
| Plaque presence | 1134 (91) | 1335 (82) | 2105 (85) | 364 (92) | 2035 (84) | 389 (93) |
| Plaque score | 3 (2, 5) | 2 (1, 3) | 2 (1, 4) | 3 (2, 5) | 2 (1, 4) | 3 (2, 4) |
| Myocardial infarction | 169 (13) | 60 (4) | 178 (7) | 47 (11) | 154 (6) | 64 (15) |
| SAF (A.U.) | 2.52 ± 0.50 | 2.31 ± 0.46 | 2.37 ± 0.48 | 2.57 ± 0.52 | 2.37 ± 0.48 | 2.54 ± 0.52 |

Abbreviations: CKD, chronic kidney disease; HDL, high-density lipoprotein; eGFR, estimated glomerular filtration rate; CAC, coronary artery calcification score; EBT, electron-beam tomography; MDCT, multi-detector computed tomography; SAF, skin autofluorescence; A. U., arbitrary unit.

**Table S3 Association between SAF and the severity of carotid plaques in the total population**

|  | **n/N, %** | **OR (95% CI)** | | | |
| --- | --- | --- | --- | --- | --- |
|  |  | **Crude model** | **Model 1** | **Model 2** | **Model 3** |
| Plaque |  |  |  |  |  |
| Absence | 413/2882, 14.3 | 1.00 (reference) | 1.00 (reference) | 1.00 (reference) | 1.00 (reference) |
| Mild | 494/2882, 17.1 | 1.55 (1.15, 2.08) | 1.28 (0.94, 1.74) | 1.27 (0.93, 1.72) | 1.17 (0.85, 1.60) |
| Moderate | 561/2882, 19.5 | 1.35 (1.01, 1.81) | 0.96 (0.71, 1.3) | 0.95 (0.7, 1.29) | 0.87 (0.64, 1.20) |
| Severe | 1414/2882, 49.1 | 3.13 (2.43, 4.04) | 1.73 (1.32, 2.27) | 1.69 (1.28, 2.23) | 1.45 (1.09, 1.93) |

Abbreviations: SAF, skin autofluorescence; n: number of participants in the group; N, number of participants with available data on plaque severity; CI, confidence interval.

ORs and 95% CIs are adjusted odds ratios and 95% confidence intervals of plaque severity relative to absence of plaques for one unit higher of SAF from the multinomial logistic regression models.

Crude model: was not adjusted for covariates.

Model 1 was adjusted for age, sex, RS subcohorts.
Model 2 was adjusted for age, sex, RS subcohorts, body mass index, dyslipidemia, and hypertension.

Model 3 was adjusted for age, sex, RS subcohorts, body mass index, dyslipidemia, hypertension, smoking status, diabetes, and eGFR.

**Table S4 Associations between SAF and endophenotypes reflecting atherosclerosis or arterial stiffness after excluding individuals with a history of myocardial infarction**

| **Outcome^a^** | **N** | **Crude model** | **Model 1** | **Model 2** | **Model 3** |
| --- | --- | --- | --- | --- | --- |
|  |  | **Odds ratio (95% CI)**^b^ | | | |
| Hypertension | 2766 | 1.59 (1.33, 1.9) | 1.08 (0.89, 1.31) | 1 (0.81, 1.22) | 0.98 (0.8, 1.21) |
| Plaque presence | 2661 | 2.1 (1.64, 2.68) | 1.34 (1.03, 1.74) | 1.32 (1.01, 1.71) | 1.17 (0.89, 1.53) |
| Categorical CAC (EBT) | 408 | 1.61 (1.06, 2.43) | 1.61 (1.06, 2.43) | 1.61 (1.06, 2.43) | 1.61 (1.06, 2.43) |
| Categorical CAC (MDCT) | 284 | 2.19 (1.35, 3.54) | 2.19 (1.35, 3.54) | 2.19 (1.35, 3.54) | 2.19 (1.35, 3.54) |
|  |  | **Beta coefficients (95% CI)**^c^ | | | |
| IMT (max) | 2657 | 0.4 (0.33, 0.48) | 0.11 (0.04, 0.18) | 0.09 (0.02, 0.16) | 0.07 (-0.01, 0.14) |
| CAC (EBT) | 408 | 0.9 (0.45, 1.34) | 0.59 (0.13, 1.05) | 0.46 (0, 0.91) | 0.36 (-0.1, 0.81) |
| CAC (MDCT) | 284 | 0.98 (0.43, 1.53) | 0.69 (0.15, 1.23) | 0.61 (0.08, 1.14) | 0.52 (-0.01, 1.06) |
| PWV | 2200 | 0.39 (0.31, 0.48) | 0.12 (0.03, 0.2) | 0.09 (0.01, 0.17) | 0.09 (0.01, 0.17) |

Abbreviations: SAF, skin autofluorescence; CI, confidence interval; IMT, carotid intima-media thickness; CAC, coronary artery calcification score; EBT, electron-beam tomography; MDCT, multi-detector computed tomography; PWV, pulse wave velocity.

a: z-scores of SBP, DBP, IMT, PWV, categorial CAC scores (≥100 vs. <100), and log transformed (CAC +1) were used in the analyses.

b: Odds ratios (95% CIs) were adjusted odds ratios associated with one-unit higher SAF.

c: Coefficients (95% CIs) were adjusted differences of the endophenotypes associated with one-unit higher SAF, in folds of SD for IMT, PWV, and in natural logarithm of (CAC +1).

Crude model: was not adjusted for covariates.

Model 1was adjusted for age (SAF), sex and RS subcohorts.

Model 2 was adjusted for BMI, dyslipidemia, and hypertension in addition to model 1.

Model 3 was adjusted for smoking status, diabetes, and eGFR in addition to model 2.

Hypertension was not adjusted for the association between SAF and hypertension.

**Figure S1 Participants inclusion and exclusion flowchart and analysis scheme**


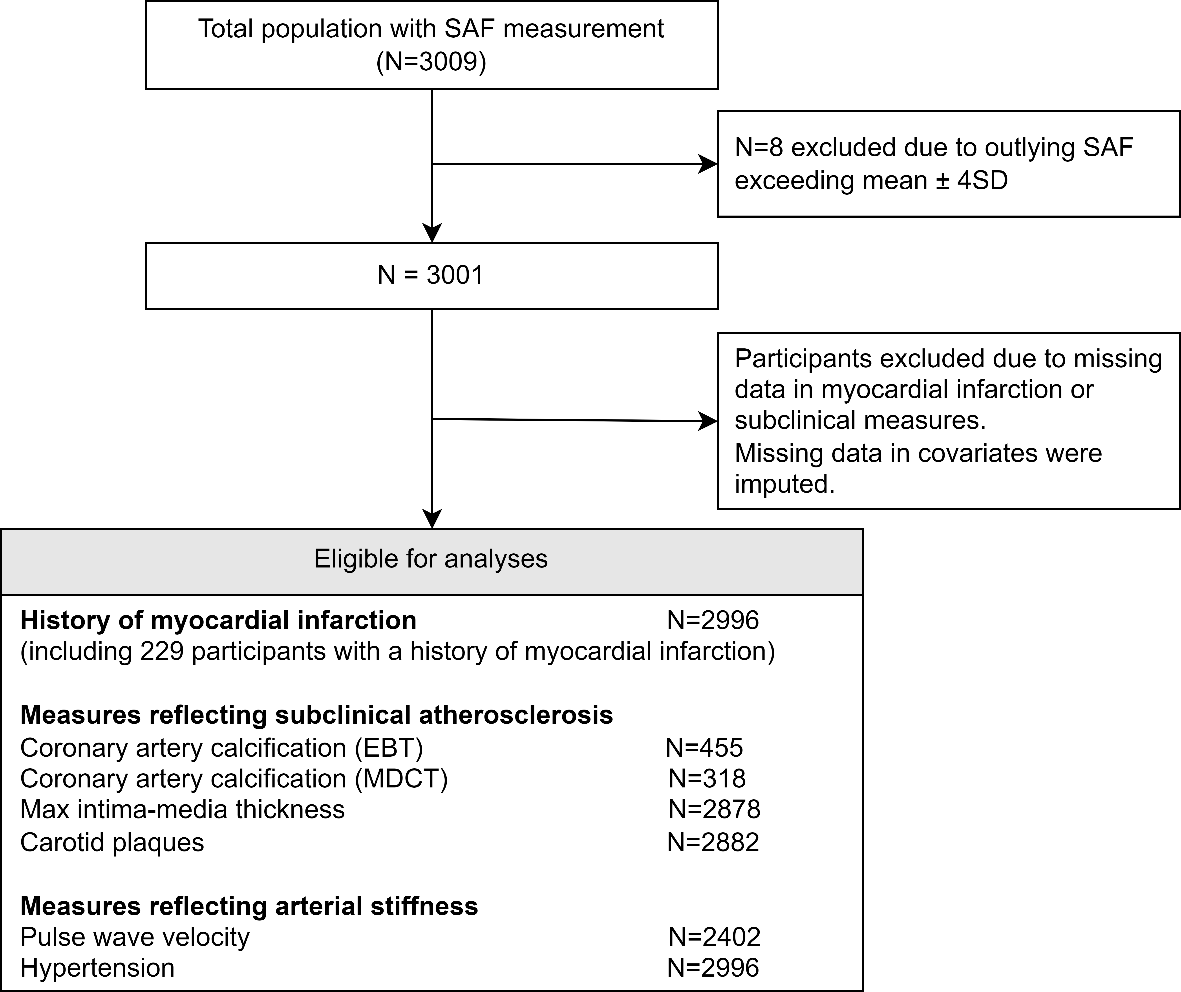


Abbreviations: SAF, skin autofluorescence; EBT, electron-beam tomography; MDCT, multi-detector computed tomography.

**Figure S2 Timeline of data collection**


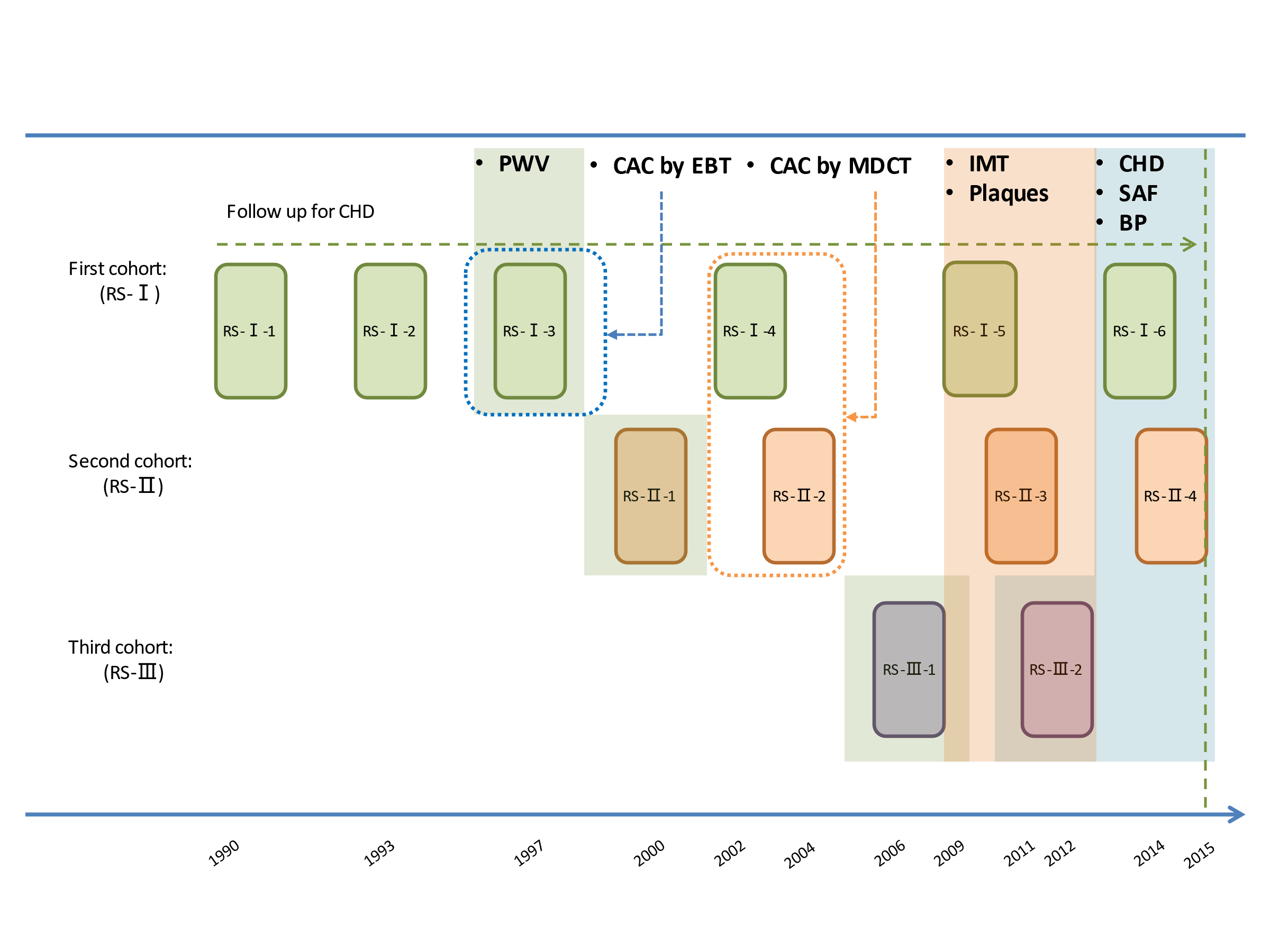


Abbreviations: RS, Rotterdam Study; CHD, coronary heart disease; PWV, pulse wave velocity; CAC, coronary artery calcification; IMT, carotid intima-media thickness; SAF, skin autofluorescence; BP, blood pressure.

**Figure S3 The directed acyclic graph for confounder selection**


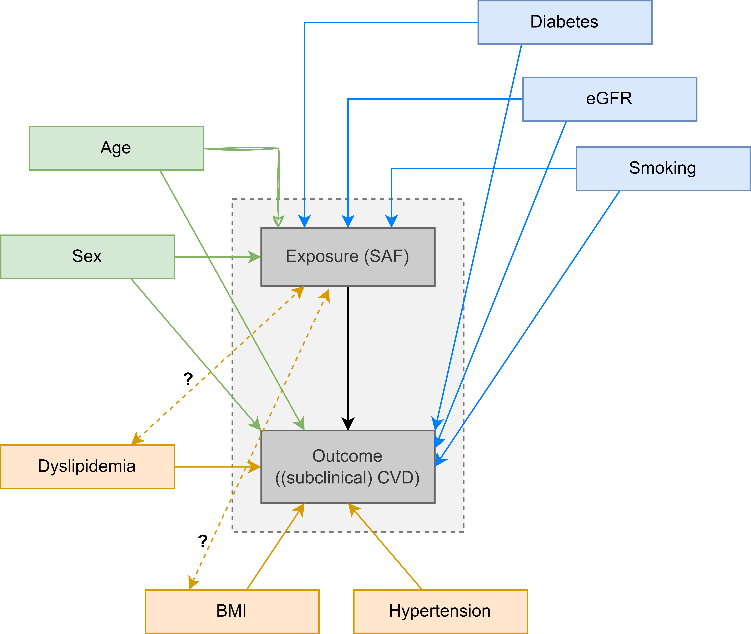


Abbreviations: SAF, skin autofluorescence; CVD, cardiovascular diseases; eGFR, estimated glomerular filtration rate; BMI, body mass index.

**Figure S4 Forest plot for the association between conventional cardiovascular risk factors and SAF**


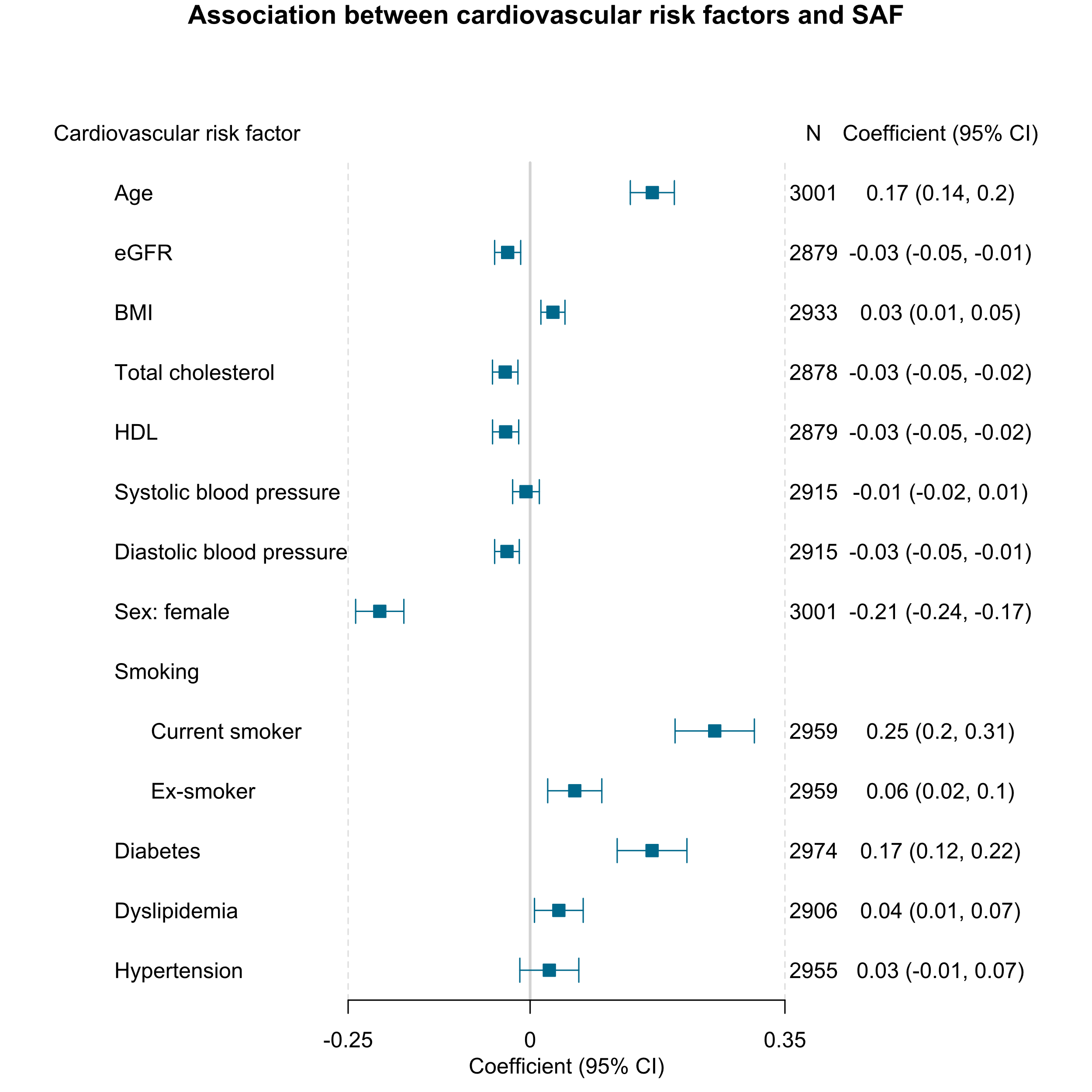


Notes:

Abbreviations: SAF, skin autofluorescence; N, number of participants with data available; CI, confidence interval; eGFR, estimated glomerular filtration rate; BMI, body mass index; HDL, high-density lipoprotein; SBP, systolic blood pressure; DBP, diastolic blood pressure.

Coefficients (95% CIs) are adjusted differences of SAF associated with one SD higher the value of continuous risk factors or the presence of binary risk factors.

a. Age was measured during SAF visits, and the other risk factors were measured during RS-Ⅰ-5th, RS-Ⅱ-3rd, and RS-Ⅲ-2nd visits.

b. For continuous cardiovascular risk factors, z-scores were used in the analyses. All the associations were adjusted for age, sex, and Rotterdam Study subcohorts, except for when the associations between age, sex, and SAF were studied.

c. One outlying value in total cholesterol was excluded from the analysis on the association between total cholesterol and SAF.

**Figure S5 The association between SAF and myocardial infarction history by the presence of diabetes or not**

n/N: number of prevalent myocardial infarction/ number of participants in the group.

ORs and 95% CIs are adjusted odds ratios and the respective 95% confidence intervals of the presence of myocardial infarction associated with one unit higher SAF.

The association was adjusted for age, sex, RS subcohorts, body mass index, dyslipidemia, hypertension, smoking status, diabetes, and eGFR. Diabetes was not adjusted in analysis stratified by diabetes status.

**Figure S6 The association between SAF and carotid plaques in men and women**


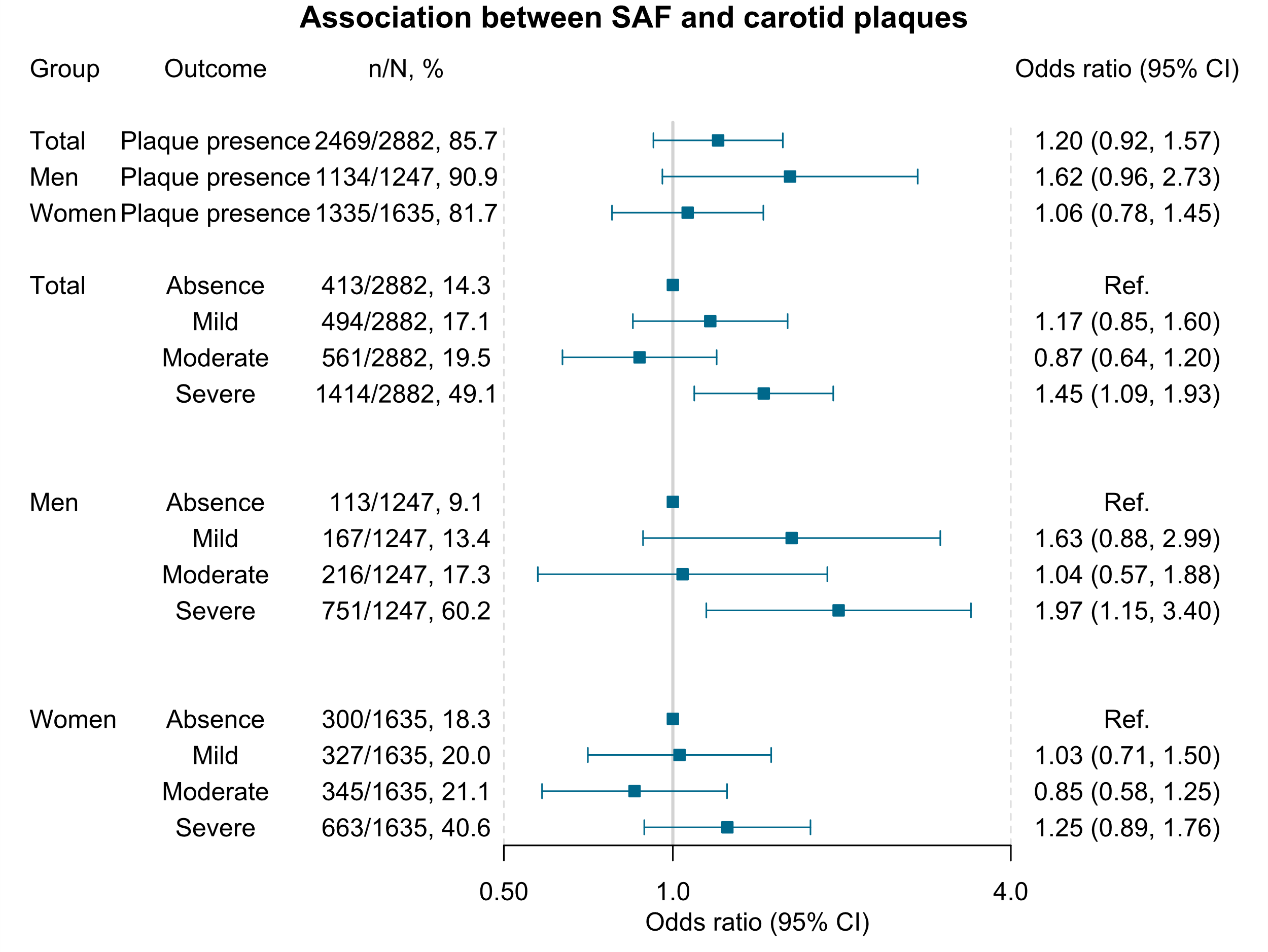


Abbreviations: SAF, skin autofluorescence; n: number of participants in the group; N, number of participants with available data on plaque severity; CI, confidence interval.

ORs and 95% CIs are adjusted odds ratios and 95% confidence intervals of plaque presence/ severity relative to absence of plaques for one unit higher of SAF from the binary or multinomial logistic regression models. The associations were adjusted for age, sex, RS subcohorts, body mass index, dyslipidemia, hypertension, smoking status, diabetes, and eGFR. Sex was not adjusted for in stratified analysis by sex.

**Figure S7 The association between SAF and pulse wave velocity by the presence of diabetes or not**


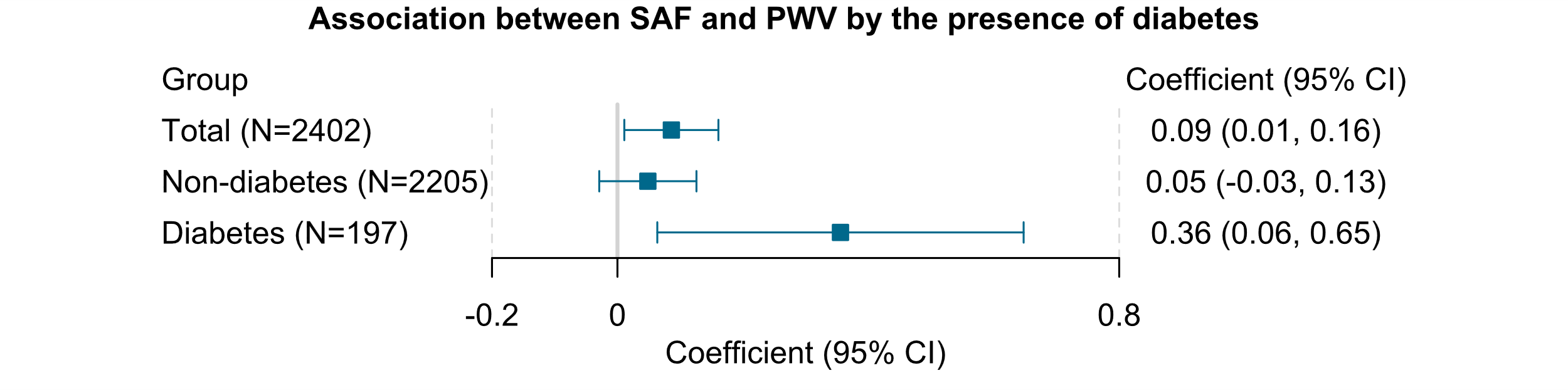


Abbreviations: SAF, skin autofluorescence; PWV, pulse wave velocity; N, number of participants included in the analysis; CI, confidence interval.

z-scores of PWV was used in the analyses.

Coefficients (95% CIs) were differences of PWV in folds of SD associated with one-unit higher SAF, adjusting for age, sex, RS subcohorts. BMI, dyslipidemia, hypertension, smoking status, diabetes, and eGFR. Diabetes was not adjusted for in stratified analysis.
